# Molecular characterization of multidrug resistant strains of *Acinetobacter baumannii* isolated from pediatric intensive care unit in a Chinese tertiary hospital

**DOI:** 10.1186/s12879-018-3511-0

**Published:** 2018-12-04

**Authors:** Yili Chen, Lu Ai, Penghao Guo, Han Huang, Zhongwen Wu, Xiaoling Liang, Kang Liao

**Affiliations:** 1 Department of Laboratory Medicine, The First Affiliated Hospital of Sun Yat-sen University, Guangzhou, 510080 Guangdong China; 2 Gaoyao People’s Hospital, Zhaoqing, 526040 Guangdong China

**Keywords:** Multidrug-resistant, *Acinetobacter baumannii*, Pediatric intensive care unit, Carbapenemase enzymes, Efflux pump

## Abstract

**Background:**

*Acinetobacter baumannii* is a nosocomial pathogen which is reported as a major cause of morbidity and mortality in intensive care units (ICUs). However, there is a lack of analysis focused on multidrug-resistant *Acinetobacter baumannii* (MDRAB) infection among patients from pediatric intensive care unit (PICU) in China. The aim of this study was to investigate the molecular characterization of MDRAB isolated from PICU.

**Methods:**

In this study, 86 isolates of MDRAB were collected from PICU patients, from the First Affiliated Hospital of Sun Yat-sen University. The minimal inhibitory concentrations (MICs) of the isolates against common antibiotics were determined. The carbapenemase-encoding resistance genes and *AdeABC-AdeRS* efflux system genes of these isolates were detected by PCR. Real-time PCR was performed to determine the relative expression of the relevant efflux pumps.

**Results:**

Among 86 strains of MDRAB, 76.7% (66/86) were carbapenem-resistant *A. baumannii* (CRAB). All 86 clinical isolates possessed the *bla*_*OXA-51*_ gene. *Bla*_*OXA-23*_ was detected as the second most frequent (90.7%) carbapenemase. Harboring *AdeABC* efflux pump genes was prevalent among the majority of the MDR isolates. Specially, the distributions of *AdeABC-AdeRS* efflux system genes in CRAB strains reached up to 90.0%. Compared with those of the CSAB strains, there was a statistically significant increasing distribution of the regulator *AdeR* and *AdeS* genes(*p < 0.05*). Moreover, CRAB strains showed significantly increased expression of *AdeB*(12.3- fold), but decreased expression of *AdeR* (3.3- fold)(*p < 0.05*).

**Conclusion:**

The present study showed a high distribution of multiple genes, mainly the genes of *bla*_*OXA-23*_*/bla*_*OXA-51*_ carbapenemase and *AdeABC* efflux pump, is responsible to distinct drug-resistance in PICU. It is urgent to strengthen the molecular epidemiological surveillance of pediatric MDRAB isolates to prevent further outbreaks. This study is of significant help for the clinicians to make therapeutic decisions and manage infection control in PICU.

## Background

*Acinetobacter baumannii* has been reported as an epidemiologically and clinically life-threatening nosocomial pathogen causing critical morbidity and mortality, especially in intensive care units (ICUs) [1]. The drug resistance rate against carbapenems of this organism is alarmingly high. It is extensively reported that carbapenem resistant strains of *Acinetobacter baumannii* (CRAB) have caused the hospital outbreaks worldwide [2–4].

Although, *A. baumannii* was frequently and classically reported as a main hospital-acquired pathogen in adults, it is also a critical pathogen in the pediatric intensive care unit (PICU), because of invasive surgery, severe basic diseases, immunodeficiency, and prolonged hospitalization among the pediatric patients [5, 6]. Several outbreaks of carbapenem-resistant *A. baumannii* in the PICU have been documented in the last decade [7–9].

To increase awareness and improvement of epidemiological surveillance is a significant and critical factor of successful infection control and is particularly recommended in ICUs. To our knowledge, few studies on the molecular characterization of MDRAB isolated from PICU are reported in China. Thus, the present study was conducted to determine the molecular characteristics among the MDRAB strains from PICU in a tertiary hospital in Guangzhou, China, during a 5-year period from January 2013.

## Methods

### Isolation and identification of bacterial strains

Eighty-six non-repetitive multidrug-resistant *Acinetobacter baumannii* strains were obtained from different PICU patients, in the First Affiliated Hospital of Sun Yat-sen University, Guangzhou, China, from January 2013 to December 2017. The isolates were collected from cultures of respiratory tract specimens (80 isolates, including 74 isolates from sputum samples and 6 isolates from nasal secretion samples), blood (5 isolates), and stool (1isolate). These strains were identified by an automated microbiology analyzer (bioMérieux, Marcy l’Etoile, France) according to the manufacturer’s instructions, and confirmed on *bla*_*OXA-51*_ polymerase chain reaction (PCR). This report was approved by the Clinical Research and Ethics Committee of the First Affiliated Hospital of Sun Yat-sen University.

### Antimicrobial susceptibility testing

Antimicrobial susceptibilities for isolates were determined initially using Gram-negative susceptibility (GNS) cards on the Vitek system (bioMérieux, Marcy l’Etoile, France) to designate isolates as either carbapenem-resistant *A. baumannii* (CRAB)(with imipenem MICs≥8 μg/ml) or carbapenem -susceptible *A. baumannii* (CSAB) (with imipenem MICs≤2 μg/ml). Susceptibility interpretations were based on CLSI clinical breakpoints (2017; CLSI Document M100-S27). The tested agents included ampicillin/sulbactam, piperacillin/tazobactam, cefotaxime, ceftazidime, cefepime, imipenem, amikacin, gentamicin, ciprofloxacin, levofloxacin, trimethoprim/sulfamethoxazole, and tigecycline. Quality control for the MIC analysis was performed with *E. coli* ATCC 25922 and *P. aeruginosa* ATCC 27853.

### Identification of the drug resistance genes

Bacterial DNA was extracted from *A. baumannii* isolates by boiling. PCR was performed using TaKaRa Ex Taq (Takara Bio Inc., Otsu, Japan). All PCR primers targeting resistance genes and mobile elements used in this study are listed in Table 1. PCR was performed in a 25 μL reaction mixture containing 1 μL primer, 1 μL Taq polymerase, 3 μL DNA template, 3.0 mM MgCl2, 2.5 μL 10 × buffer, 0.2 mM dNTPs, and nuclease-free water. Amplification conditions consisted of denaturation at 94 °C for 5 min and 30 cycles of denaturation at 94 °C for 1 min, annealing at 56 °C for 30 s and extension at 72 °C for 1 min, with a final extension at 72 °C for 10 min. PCR products were detected in 2% agarose gel.

**Table 1:** Primer sequences of the target genes

Gene	Primer Sequence(5′ → 3′)	Size (bp)	Reference
IMP	F: ATGAGCAAGTTATCTGTATTCTTTAT	741	[10]
	R: TTAGTTGCTTAGTTTTGATGGTTT		
KPC	F: TCGCCGTCTAGTTCTGCTGTCCT	965	[10]
	R: CCGCGCAGACTCCTAGCCTAA		
NDM-1	F: TCACCGAGATTGCCGAGCGA	457	[10]
	R: GGGCAGTCGCTTCCAACGGT		
VIM	F: GGTCGCATATCGCAACGCAGT	636	[10]
	R: CGGCGACTGAGCGATTTTTG		
IMI	F: CCATTTCACCCATCACAAC	440	[10]
	R: CTACCGCATAATCATTTGC		
SPM	F: CTGCTTGGATTCATGGGCGC	783	[10]
	R: CCTTTTCCGCGACCTTGATC		
OXA-51	F: TCCAAATCACAGCGCTTCAAAA	639	[10]
	R: TGAGGCTGAACAACCCATCCA		
OXA-23	F: ACTTGCTATGTGGTTGCTTCTTCTT	797	[10]
	R: TTCAGCTGTTTTAATGATTTCATCA		
OXA-24	F: CAGTGCATGTTCATCTATT	702	[10]
	R: TCTAAGTTGAGCGAAAAG		
OXA-58	F: AAGTATTGGGGCTTGTGCTG	599	[10]
	R: CCCCTCTGCGCTCTACATAC		
ISAba1-OXA-51	F: CACGAATGCAGAAGTTG	1200	[10]
	R: CTTCTGTGGTGGTTGGC		
AdeA	F: GAGGTGGCAAGACTCAAAGTTC	113	[11]
	R: GCTAGAGCCTGACGATACTGAGC		
AdeB	F: TACCGGTATTACCTTTGCCGGA	250	[11]
	R: GTCTTTAAGTGTCGTAAAAGCCA		
AdeC	F: ACAATCGTATCTCGTGGACTC	361	[11]
	R: TAGAAACTGGGTTATTGGGGT		
AdeR	F: ACTACGATATTGGCGACATT	447	[12]
	R: GCGTCAGATTAAGCAAGATT		
AdeS	F: TTGGTTAGCCACTGTTATCT	544	[12]
	R: AGTGGACGTTAGGTCAAGTT		

### RNA expression of efflux pump genes

The expression of efflux pumps of *AdeABC-AdeRS* was quantified. The total RNA of isolates was extracted with TRIzol extraction (Invitrogen, Carlsbad, CA, USA) according to the manufacturer’s instructions. The relative expression of the efflux pumps was determined using real-time PCR by SYBR® Premix Ex TaqTM (Takara Bio Inc., Otsu, Japan) on the Applied Biosystems® 7500 Fast Dx Real-Time PCR Instrument (Life Technologies Corporation, Foster City, CA). The RT-PCR reaction mixture was prepared in a volume of 20 μL comprised of 3 μL of cDNA, 10 μL 2 × SYBR® Premix Ex Taq II (Tli RNaseH Plus), 0.4 μL 50 × ROX Reference Dye or Dye II and 0.8 μL of each primer. The PCR reaction was carried out under the following conditions: 1 cycle of 30 s at 95 °C, 40 cycles of denaturation at 95 °C for 30 s, annealing at 53 °C for 30 s, and elongation at 72 °C for 30 s, with one final cycle of 5 min at 72 °C. Reactions were repeated in triplicate and the fold changes in expression of these genes were calculated relative to the level of housekeeping gene 16S rDNA using the comparative C_T_ method (2^–ΔΔCT^ method).

### Statistical analysis

**Fig. 1:**
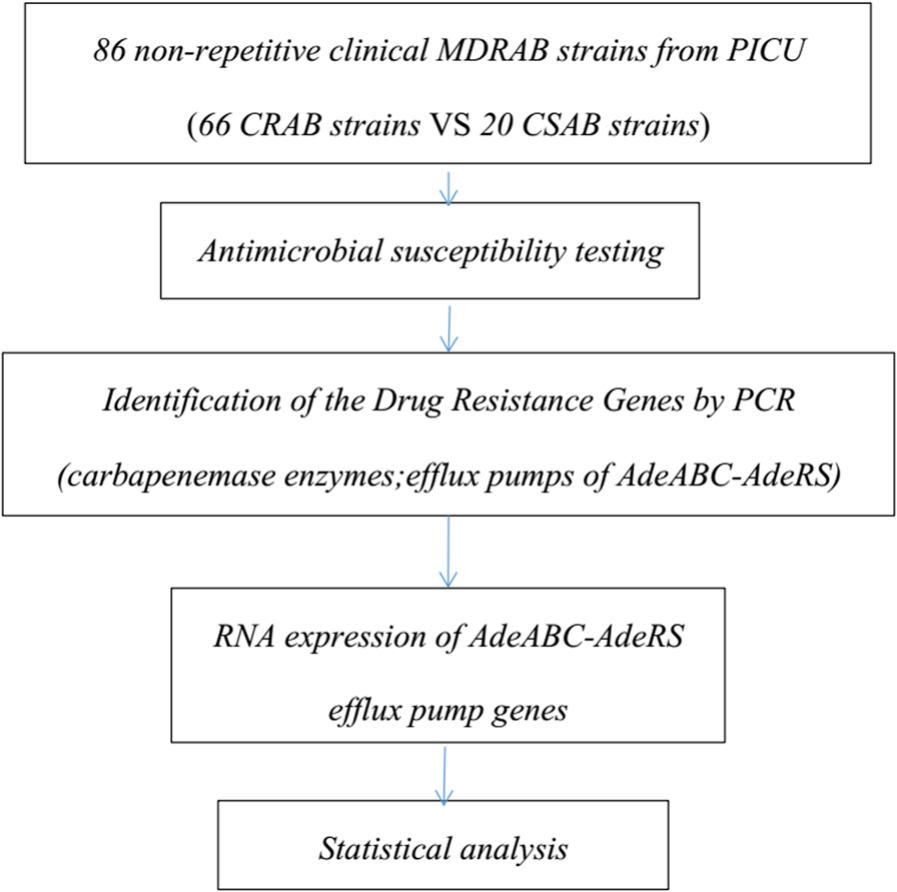
The flow chart of this research methods

Data are described using the mean and standard deviation (mean ± s). Categorical variables were compared using Fisher’s exact test. A *p* value of 0.05 was considered statistically significant. Statistical Package for the Social Sciences (SPSS) for Windows Version 16.0 (SPSS Inc.; Chicago, IL, USA) was used in this study (Fig. 1 showed a flow chart for representing this research methods).

## Results

### Antimicrobial susceptibility profile

The findings of the antibiotic susceptibility testing for all isolates were showed in Table 2. Among the 86 MDRAB strains, 76.7% (66/86) were CRAB, which showed 100% resistant to many of the tested antimicrobial agents, except tigecycline.

**Table 2:** Antimicrobial susceptibility (%) of 86 strains of MDR *Acinetobacter baumannii* isolates

Antibiotics agents	CRAB	CSAB	Total
	(*n* = 66)	(*n* = 20)	(*n* = 86)
ampicillin	0 (0/66)	0 (0/20)	0 (0/86)
ampicillin/sulbactam	0 (0/66)	0 (0/20)	0 (0/86)
piperacillin/tazobactam	0 (0/66)	80.0 (16/20)	18.6 (16/86)
cefotaxime	0 (0/66)	0 (0/20)	0 (0/86)
ceftazidime	0 (0/66)	10.0 (2/20)	2.33 (2/86)
cefepime	0 (0/66)	30.0 (6/20)	6.98 (6/86)
aztreonam	0 (0/66)	30.0 (6/20)	6.98 (6/86)
imipenem	0 (0/66)	100 (20/20)	23.3 (20/86)
amikacin	0 (0/66)	100 (20/20)	23.3 (20/86)
gentamicin	0 (0/66)	80.0 (16/20)	18.6 (16/86)
ciprofloxacin	0 (0/66)	75.0 (15/20)	17.4 (15/86)
levofloxacin	0 (0/66)	80.0 (16/20)	18.6 (16/86)
trimethoprim/sulfamethoxazole	0 (0/66)	75.0 (15/20)	17.4 (15/86)
tigecycline	100 (66/66)	100 (20/20)	100 (100/100)

### Distribution of the carbapenemase and AdeABC-AdeRS efflux system genes

The distribution of carbapenemase and efflux pump genes in drug-resistant strains was shown in Table 3. Of note, *bla*_*OXA-51*_ genes were present in all 86 MDR *A. baumannii* isolates. *Bla*_*OXA-23*_ was detected the second most frequently (90.7%) of carbapenemase. The ISAba-OXA-51, *bla*_*OXA-24*_, *bla*_*OXA-58*_, and NDM-1 genes were detected in 23.3% (20/86), 22.1%(19/86), 3.49% (3/86), and 3.49% (3/86) of the 86 MDR isolates, respectively. Among the CRAB strains, there was a statistically significant increasing distribution of *bla*_*OXA-24*_, *bla*_*OXA-58*_, and ISAba-OXA-51(*p < 0.05*). The majority of the MDR isolates were found to harbor *AdeABC* efflux pump genes. Notably, the distributions of *AdeABC-AdeRS* efflux system genes among CRAB strains were more than 90.0%, and compared with the CSAB strains, there was a statistically significant increasing distribution of the regulator *AdeR* and *AdeS* genes(*p < 0.05*). Specifically, there was a higher distribution of co-harboring *AdeABC-AdeRS* efflux system genes and *bla*_*OXA-23*_ among the CRAB strains (Table 4).

**Table 3:** Distribution of *Carbapenemase* genes and *Ade* efflux pump genes in the 86 MDRAB isolates in this study

Gene	CRAB (*n* = 66)		CSAB (*n* = 20)		*P value*	Total (*N*/%)
	*N*	Prevalence rate	*N*	Prevalence rate		
IMP	0	0	0	0	–	0
KPC	0	0	0	0	–	0
NDM-1	3	4.54	0	0	0.52	3 (3.49)
VIM	0	0	0	0	–	0
IMI	0	0	0	0	–	0
SPM	0	0	0	0	–	0
OXA-51	66	100	20	100	–	86 (100)
OXA-23	60	90.9	18	90.0	0.92	78 (90.7)
OXA-24	19	28.8	0	0	0.02	19 (22.1)
OXA-58	3	4.54	0	0	0.07	3 (3.49)
ISAba-OXA-51	20	30.3	0	0	0.01	20 (23.3)
AdeA	61	92.4	18	90.0	0.71	79 (91.9)
AdeB	65	98.5	18	90.0	0.54	83 (96.5)
AdeC	61	92.4	15	75.0	0.10	76 (88.4)
AdeR	60	90.9	10	50.0	0.04	70 (81.4)
AdeS	61	92.4	11	55.0	0.04	72 (83.7)

**Table 4:** The distribution of co-harbouring genes among the 86 MDRAB strains

Co-harbouring gene	CRAB (*n* = 66)		CSAB (*n* = 20)			Total
	*N*	Prevalence rate	*N*	Prevalence rate	*P value*	(*N*/%)
AdeABC+blaOXA23	61	92.4	15	75	0.032	76 (88.4)
AdeABC-RS + blaOXA23	60	85.7	10	50	0.048	70 (81.4)

### Relative expression levels of the AdeABC-AdeRS efflux system genes

The level of expression of the *AdeABC-AdeRS* efflux system genes was measured by qRT-PCR (Table 5). It was found that, compared with those of CSAB strains, CRAB strains showed significantly increased expression of *AdeB*(12.3- fold), but decreased expression of *AdeR* (3.3- fold). However, there was no significant difference of the relative expression of *AdeA*, *AdeC* or *AdeS* between the two groups.

**Table 5:** Relative expression levels of the *AdeABC-AdeRS* efflux system genes in MDR *Acinetobacter baumannii* isolates

Efflux system gene	MDRAB(*n* = 86)	
	CRAB	CSAB
	(*n* = 66)	(*n* = 20)
*AdeA*	2.07 ± 0.88	2.13 ± 0.35
*AdeB*	2.83 ± 0.39*	0.23 ± 0.50
*AdeC*	1.77 ± 0.67	1.66 ± 1.21
*AdeR*	1.27 ± 1.01*	4.23 ± 1.52
*AdeS*	1.80 ± 2.50	1.12 ± 1.24

*p < 0.05

## Discussion

In this study, we found there was a 76.7% prevalence of imipenem-resistance among the MDRAB in PICU from our hospital. Tigecycline proved to be the most active antibiotics. Even though effective and safe therapeutic options are limited against MDRAB strains to PICU patients, combination therapy is still the potential choice to fight against carbapenem resistance in MDRAB. Polymyxins, tigecycline, amikacin, imipenem, meropenem, ceftazidime, sulbactam-based regimens etc. are commonly considered. It was reported that most patients received with a combination of β-lactams and aminoglycoside, for example, imipenem-amikacin for initial empirical antibiotic therapy on MDRAB [10]. The clinical application of tigecycline is accumulating in the treatment of MDRAB infections patients. Although many researches including this present study showed high in-vitro susceptibility rates, the role of tigecycline in treating MDRAB infections remains controversial [11]. Some of studies challenged the use of a tigecycline-based regimen in the treatment of MDRAB infections, because of their association higher hospital mortality, lower microbial eradication rates and longer hospital stay.

Drug resistance mechanisms of MDRAB are sophisticated, including the production of enzymes that act as intrinsic drug resistance, the pathogen’s amazing ability to obtain resistance determinants horizontally, accident changes of outer membrane permeability, and the role of efflux systems [12, 13]. For example, mainly due to producing various carbapenemase enzymes such as class B metallo-β -lactamases (MBLs) and class D oxacillinases (OXAs), *A. baumannii* developed its resistance to carbapenems.

The substrate specificities of the OXA-type carbapenemases are various, but usually the enzymes hydrolyse penicillins and the narrow-spectrum cephalosporins, cefalotin and cefaloridine more efficiently, than the extended-spectrum-β -lactams. All OXA-type carbapenemases can be inhibited better by tazobactam. Although the catalytic efficiency of OXA-carbapenemases for the hydrolysis of carbapenems is lower than that of MBL (100–1000 times lower), it is important to consider them as potential hazards because their expression can be regulated by upstream insertion of IS elements such as *ISAba1*. [14] *Bla*_*OXA-23*_, *bla*_*OXA-24*_ and *bla*_*OXA-58*_ are most common OXA-type genes detected within clinical MDRAB isolates, typically among Asia-Pacific region[15–18]. It is reported that the *bla*_*OXA-23*_ gene is much more prevalent, while the *bla*_*OXA-58*_ gene was rarely found in MDRAB isolates [18]; in addition, MBLs SIM, IMP, and VIM-producing *A. baumannii* isolates are also worth noting [19].

In our study, all of the 86 clinical isolates possessed the chromosomally encoded enzyme gene *bla*_*OXA-51*_. Among these MDR isolates, such a high frequency of *bla*_*OXA-51*_ gene detected is likely to suggest that an intrinsic drug resistance mechanism plays an important role on the multidrug resistance. However, further quantitative assay is necessary to value the contribution of *bla*_*OXA-51*_ gene in the mechanism of multidrug resistance of these isolates.

The widespread prevalence of *bla*_*OXA-23*_ gene in *Acinetobacter spp.* has been recently reported, that the plasmids pAZJ221 and Tn2009 were likely to play a key role on horizontally transferring *bla*_*OXA-23*_ gene [20]. Hu et al. investigated 17 cases of *Acinetobacter baumannii* infection in a neonatal intensive care unit (NICU), and *bla*_*OXA-23*_ was detected in all of the isolates, suggesting that the detection of the *bla*_*OXA-23*_ gene might be of significant help in neonatology decision-making on managing nosocomial infection [21]. Consistent with these findings, the present study showed that *bla*_*OXA-23*_ was detected most frequently (90.7%) of carbapenemase among the 86 MDRAB from PICU. Notably, we showed 18 strains among the 20 CSAB strains displayed imipenem susceptibility.To our knowledge, the OXA-23 enzyme has a high affinity to hydrolyse imipenem, however, there have been reports on occurrence of *bla*_*OXA-23*_ gene in imipenem-susceptible *Acinetobacter baumannii* [22]. Imipenem susceptibility may be explained by the absence of the *ISAba1* element upstream of the *bla*_*OXA-23*_ gene, since *ISAba1* has promoter sequences to up-regulate *bla*_*OXA-23.*_ Therefore, the *bla*_*OXA-23*_ gene may be silently transmitted in hospital settings, highlighting the undetected threat to the carbapenemase gene pool.

In addition to the production of carbapenemase, efflux pumps systems also have presented important roles in the carbapenem resistance of MDRAB to antibiotics, especially the presence of *AdeABC* multidrug efflux pump [23–25]. Reports from other studies had indicated that the expression of *AdeABC* efflux pumps were obviously influenced by *AdeRS* [26, 27]. Either the point mutations in *AdeRS* or by the insertion sequence (IS) Aba-1 insertion upstream of the *AdeABC* operon would result in over-expression of the *AdeABC* efflux pump [28]. Remarkably, single point mutations in *AdeS* (Thr153Met) and *AdeR* (Pro116Leu) are believed to be in a close relationship with *AdeABC* over-expression [29], and, as a result, these changes would lead to resistance against lots of antibiotics, including β-lactams, chloramphenicol, tetracyclines, aminoglycosides, and fluoroquinolones [30].

In the present study, the *AdeABC-AdeRS* efflux systems were widely distributed among these isolates. Specifically, in the 66 CRAB strains, the distributions of *AdeABC-AdeRS* efflux system genes were more than 90.0%. The CRAB group had a statistically significant increased expression of *AdeB* gene, but decreased expression of *AdeR*, but there was no significant difference of the relative expression of *AdeS*. These results indicated the *AdeABC-AdeRS* efflux systems might be potential cause of carbapenem resistance, with the over-expression of *AdeB* gene. Moreover, the down-regulated expression of *AdeR* gene might contribute to this process. Though our study did not show significant different expression on *AdeS* between the two groups, the research by Srinivasan et al [29] has displayed that *AdeS* could confer resistance to some antibiotics. Since bacterial isolates collected in the present study were from a single clinical center, further investigation about the role of *AdeS* gene on carbapenem resistance is warranted.

Studies about *A.baumannii* isolates co-harboring oxacillinase and efflux pump genes are accumulating. For example, in a hospital of Korea, it is proved that the expression both of *bla*_*OXA-23*_ gene and *AdeABC* efflux pump genes were responsible for acquiring carbapenem resistant MDRAB isolates [31]. Hu et al. have indicated that among the imipenem resistant *A. baumannii* isolates, the production of carbapenemase carrying *bla*_*OXA-51*_*/bla*_*OXA-66*_ genes could contribute to the intrinsic resistance to imipenem, but the drug efflux pump systems were much more responsible for the widespread dissemination of imipenem-resistant *A. baumannii* [32]. It is believed that the OXA-type carbapenemases can be fortified when increased expression of efflux pumps are present.

Our study had the following limitations. First, the isolates were collected from one centre, and the sample size was small, which might result in a special tendency of the molecular characteristics of MDRAB. Second, except *AdeABC*, other efflux systems have not been investigated in the present study. Further studies on how the *AdeABC-RS* system influences the decreasing carbapenem-susceptibility against MDRAB are needed.

## Conclusion

The present study showed high distributions of multiple genes, mainly the genes of *bla*_*OXA-23*_*/bla*_*OXA-51*_ carbapenemase and *AdeABC* efflux pump system, indicating a potential threat of MDRAB in PICU. Therefore, to develop an effective guidance to prevent hospital-acquired infections caused by MDRAB in ICUs is critical. All-around interventions such as hand hygiene, environmental cleaning, contact isolation precautions, and active surveillance are significantly important to reduce the incidence of *A. baumannii* infections.

## Abbreviations

CRAB: Carbapenem-resistant *A. baumannii*
CSAB: Carbapenem -susceptible *A. baumannii*
MBLs: Metallo-β -lactamases
MDRAB: Multidrug-resistant *Acinetobacter baumannii*
MICs: Minimal inhibitory concentrations
OXAs: Oxacillinases
PCR: Polymerase chain reaction
PICU: Pediatric intensive care unit
